# Improving the Endosomal Escape of Cell-Penetrating Peptides and Their Cargos: Strategies and Challenges

**DOI:** 10.3390/ph5111177

**Published:** 2012-11-01

**Authors:** Alfredo Erazo-Oliveras, Nandhini Muthukrishnan, Ryan Baker, Ting-Yi Wang, Jean-Philippe Pellois

**Affiliations:** Department of Biochemistry and Biophysics, Texas A&M University, Room 430, 300 Olsen Blvd., College Station, TX 77843-2128, USA; Email: alfredo_erazooliveras@tamu.edu (A.E.O.); nandhini.muthukrishnan@tamu.edu (N.M.); ryanbaker712@gmail.com (R.B.); tywangtw@tamu.edu (T.Y.W.)

**Keywords:** endosomal escape, cell-penetrating peptide, photochemical internalization, multivalent peptides, pH-dependent membrane-active peptides

## Abstract

Cell penetrating peptides (CPPs) can deliver cell-impermeable therapeutic cargos into cells. In particular, CPP-cargo conjugates tend to accumulate inside cells by endocytosis. However, they often remain trapped inside endocytic organelles and fail to reach the cytosolic space of cells efficiently. In this review, the evidence for CPP-mediated endosomal escape is discussed. In addition, several strategies that have been utilized to enhance the endosomal escape of CPP-cargos are described. The recent development of branched systems that display multiple copies of a CPP is presented. The use of viral or synthetic peptides that can disrupt the endosomal membrane upon activation by the low pH of endosomes is also discussed. Finally, we survey how CPPs labeled with chromophores can be used in combination with light to stimulate endosomal lysis. The mechanisms and challenges associated with these intracellular delivery methodologies are discussed.

## 1. Introduction

Cell-penetrating peptides (CPPs) possess the ability to carry cell-impermeable molecules inside live cells [1,2,3]. One of the first CPPs that was reported to display cellular transduction activity was TAT, a peptide derived from the HIV-1 TAT protein [4]. Over the past two decades, several peptide sequences have been shown to exhibit transduction activity. These peptide sequences include penetratin, a peptide derived from the *Drosophila antennapedia* homeoprotein, the VP22 peptide from the herpes simplex virus VP22 protein, the chimeric peptide transportan (TP), and the synthetic polyarginine peptides [5,6,7,8]. Other well-known CPPs include amphipathic peptides such as MPG, Pep-1, MAP, and PPTG1 [9,10,11]. CPPs have been shown to cause cellular uptake of cargos such as DNA, siRNA, proteins, fluorophores, and drugs both *in vivo* and *in vitro* [12,13,14,15,16,17,18].

**Figure 1:**
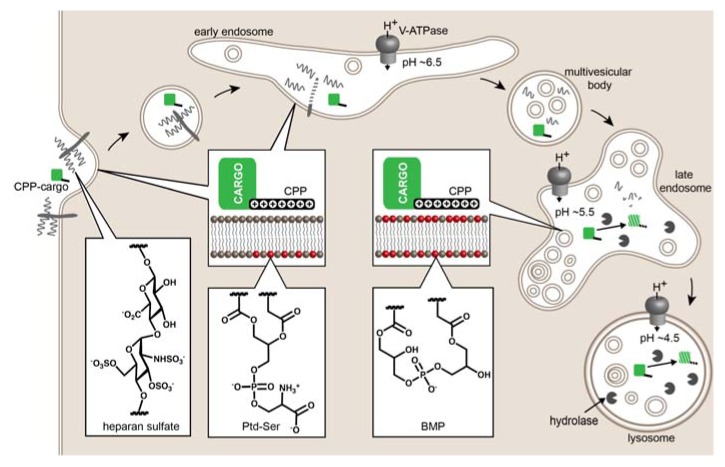
Model of the trafficking of a CPP-cargo conjugate through the endocytic pathway. From left to right: A CPP-cargo binds to HSPGs on the cell surface and induces endocytosis. Endocytosis leads to uptake and entrapment of the CPP-cargo inside an endocytic vesicle. The endosomal membrane contains the lipid phosphatidylserine (Ptd-Ser) in its outer leaflet. During endosomal maturation, a vacuolar H^+^-ATPase acidifies the lumen of endocytic organelles. The CPP-cargo reaches early endosomes (pH~6.5). Concurrently, hydrolases partially degrade HSPGs and release HS fragments. Upon further maturation, the CPP-cargo reaches multivesicular bodies, late endosomes (pH~5.5), and lysosomes (pH~4.5). The membrane of the intraluminal vesicles of late endosomes is enriched with BMP. HS is further degraded to smaller fragments. The CPP-cargo is susceptible to degradation due to the low pH and lysosomal hydrolases.

Certain CPPs, when attached to small cargos, can directly translocate across the plasma membrane of cells [19]. However, when conjugated to macromolecules or when used at low concentrations, CPPs enter cells using the endocytic pathway [20,21,22,23]. CPPs can hijack or induce one or more endocytic mechanisms and, as a result, CPP-cargos tend to rapidly accumulate inside endocytic organelles (Figure 1) [24,25]. Many reports have now established that CPP-cargos can then escape from endocytic organelles to reach the cytosolic space of cells [26]. CPPs therefore appear to promote the release of molecules trapped inside endosomes and this activity is essential for successful intracellular delivery. As a matter of fact, cargos that remain entrapped within endosomes cannot display biological activity since they cannot reach their cytosolic targets [27]. In addition, macromolecules are subjected to degradation by acidic pH or hydrolases as they traffic into late endosomes or lysosomes (Figure 1) [28].

Although CPP mediated endocytic uptake is typically efficient, it appears that the endosomolytic activity of CPPs is, in contrast, very poor [29]. For instance, CPPs conjugated to small organic fluorophores or fluorescent proteins display a punctate distribution inside cells consistent with endosomal entrapment when observed by fluorescence microscopy [30,31,32]. Often, no cytosolic signal is observed, indicating that the CPP-cargo has not reached the cytosol [33]. Consistent with this notion, CPPs conjugated to biologically active cargos often fail to display significant cellular activities [34]. (In contrast, reagents that disrupt endosomes have been shown to dramatically improve the cellular delivery and biological activity of CPP-cargos [35].) In order to optimize delivery, a current challenge is therefore to increase the endosomolytic activity of CPPs. 

Herein, we review the evidence for CPP-mediated endosomal escape and examine several mechanisms that have been proposed for this activity. We also survey several strategies that have been pursued to increase the endosomal escape of CPP-cargos. We describe in particular the incorporation of lytic moieties triggered by pH or light as well as the oligomerization of CPPs.

## 2. Evidence of CPP-Mediated Endosomal Escape

The mechanisms by which CPP-cargos enter cells remain a matter of debate. There are, in particular, two general views on how a CPP-cargo conjugates might reach the cytosolic space of cells. Several lines of evidence support the notion that cellular delivery takes place by direct plasma membrane translocation [36,37]. This is a single-step process in which a CPP-cargo crosses the external lipid bilayer of a cell. This process might involve formation of inverted micelles or transient pores [38,39,40,41]. Direct translocation of fluorescent CPPs across the plasma membrane has been observed by fluorescence microscopy [42]. CPPs have also been shown to destabilize the plasma membrane and lead to the influx of molecules such as Ca^2+^ inside cells [11]. Interestingly, Ca^2+^ can trigger the plasma membrane repair response [43]. It has therefore been suggested that the plasma membrane translocation of CPPs might be masked by this membrane repair response [43]. 

Another well-established route of cellular entry for CPP-cargos is endocytosis [44]. This mechanism of cellular penetration, which is often compared to a Trojan horse approach, involves two distinct steps: endocytic uptake followed by endosomal escape (Figure 1). CPPs can be internalized by passive fluid-phase endocytosis or by binding to cell-surface components that are being internalized in a piggyback manner [45]. Additionally, several CPPs directly stimulate endocytic uptake. TAT and polyarginine peptides can, for instance, induce macropinocytosis [46,47]. The molecular details for this activity are here again a matter of debate. Heparan sulfate proteoglycans (HSPGs) have been implicated in this activity [48,49]. While positively charged CPPs undeniably interact with the negatively charged heparan sulfate (HS), the question of whether this interaction directly triggers macropinocytosis remains unanswered. In addition, other mechanisms of CPP uptake have been observed, including clathrin and caveolin-mediated endocytosis [50,51]. Overall, it should be noted that direct plasma membrane translocation and endocytosis are not mutually exclusive. Interestingly, it has been suggested that partial plasma membrane translocation of TAT might induce endocytosis. A model has been proposed where the CPP reaches the inner leaflet of the plasma membrane while the attached cargo remains trapped on the external side of the membrane. Under such circumstances, TAT has been shown to induce actin rearrangement in model systems, indicating a possible mechanism of macropinocytosis induction [52]. Regardless of the mode of uptake, it appears that in many cases, endocytosis is the prevalent gateway into cells. The most direct evidence for the importance of endocytosis comes from experiments involving the delivery of biologically active cargos. In such cases, inhibitors of endocytosis often abolish the cellular activity of the CPP-cargo. An example includes Cre recombinase conjugated to TAT. TAT-Cre conjugate is able to penetrate cells and activate expression of a luciferase or GFP reporter upon recombination of a plasmid containing the *loxP* sequence [53,54]. In the presence of amiloride and cytochalasin-D, inhibitors of macropinocytosis, activation of the reporter by TAT-Cre is greatly reduced [53]. Other examples include CPP-mediated delivery of a peptide nucleic acid (PNA) complex, which is able to induce splice correction of an aberrant splice site in a luciferase reporter gene. Here again, the CPPs TAT, penetratin, and TP appear to require endocytosis to allow delivery of the PNA. In the presence of chloroquine, a lysosomotropic agent, the splice correction efficacy increased 4-fold. On the contrary, inhibition of endocytosis by lowering the temperature to 4°C decreased the splice correction efficacy significantly [55]. These experiments reveal that direct plasma membrane translocation does not contribute significantly to delivery and that endocytosis is a required step. They also suggest that endosomal escape must take place. Indeed, material present in the lumen of endocytic organelles is topologically separate from the cytosolic space or other intracellular organelles such as the nucleus. For instance, in order for TAT-Cre to activate a reporter gene, it must gain access to the cytosol and presumably the nucleus. It must therefore escape the endocytic pathway. How and to what extent CPPs can mediate endosomal escape remains, however, unclear.

### Mechanisms of Endosomal Escape

Because endosomal release might determine the efficiency with which a cargo reaches the cytosol of cells, understanding the mechanisms involved in this step is important. To date, these mechanisms remain poorly defined. Part of the challenge associated with understanding how CPP-cargos escape from the endocytic pathway is that efficiency of endosomal escape is often poor. Cargos that typically require fewer copies to elicit a biological response certainly demonstrate that endosomal escape takes place (as is the case for TAT-Cre). However, cargos that require more copies inside cells for activity often fail to show significant biological effects. Several assays have been developed to assess the endosomal escape efficiency of CPPs. Fluorescence-based methods have revealed that CPPs conjugated to fluorescent cargos typically remain localized within the endocytic pathway [33]. Mass spectrometry approaches have also confirmed these results, though estimates vary widely depending on the conditions used [56,57,58,59,60]. Maybe the most telling examples are not those in which delivery worked, but the applications where no cytosolic delivery is detected [61,62,63,64]. For instance, TAT has been conjugated to a ubiquitin cargo designed to be cleaved by cytosolic deubiquitinating enzymes upon escape from endosomes. However, upon incubation with cells, endocytosis was detected but cleavage of ubiquitin was not [64]. It was therefore concluded that the protein did not access the cytosol to any detectable extent.

While many mechanisms have been proposed to explain plasma membrane translocation [65], fewer have been offered to explain endosomal escape. First, it should be noted that mechanisms that imply the disruption of lipid bilayers are not only applicable to plasma membrane translocation but also to endosomal escape. A mechanism that has been proposed to explain how CPPs could translocate across the plasma membrane of cells involves the negatively charged phospholipid phosphatidylserine (Ptd-Ser). Positively charged CPPs are known to bind negatively charged phospholipids such as Ptd-Ser [66,67]. This phospholipid is, however, found mainly in the inner leaflet of the plasma membrane of mammalian cells (Figure 1) [68,69]. The outer leaflet of the plasma membrane bilayer is, in contrast, composed of zwitterionic phospholipids for which CPPs have little affinity [70,71]. Nevertheless, it was proposed that CPPs and Ptd-Ser, positioned on either side of the lipid bilayer, might together form the equivalent of a membrane capacitor. This capacitor theoretically generates a membrane potential high enough to create a reversible electropore on a membrane [72]. This model also agrees with experimental data that has found a relatively enhanced ability of oligoarginine peptides to deliver cargo molecules across membranes when compared to oligolysine peptides [73]. The membrane capacitor model suggests that oligoarginines could bind the negatively charged phosphate groups in phospholipids better than oligolysines and form a more stable CPP-Ptd-Ser capacitor. Because the Ptd-Ser lipid asymmetry is presumably maintained within the endocytic pathway, such mechanisms could take place inside endosomes [68]. If this is true, a key question lies in determining how a CPP might permeabilize an endosome without acting first on the plasma membrane. A possible answer to this question could involve the difference in how CPPs access these different bilayers. As previously stated, positively charged CPPs interact with HSPGs present on the cell surface (Figure 1) [74]. The abundance of HS at the plasma membrane might therefore contribute to reducing the concentration of CPP directly in contact with lipids. During endocytosis, HSPGs are presumably internalized with CPPs (Figure 1) [30,75,76]. HSPGs therefore continue to have an inhibitory effect on the membrane disruption activity of CPPs [77]. However, HSPGs are gradually hydrolyzed during maturation within the endocytic pathway (Figure 1) [78,79]. HS hydrolysis and release from the membrane of endosomes could in turn favor the interaction between CPPs and endosomal lipid bilayers [30].

Several lines of evidence suggest that CPPs could disrupt the lipid bilayer of endocytic organelles more readily than that of the plasma membrane. For instance, positively charged CPPs preferentially bind negatively charged phospholipids over neutral ones [36,80]. Interestingly, the intraluminal lipid bilayers of late endosomes appear to be uniquely enriched in the negatively charged phospholipid bis(monoacylglycero)phosphate (BMP), also known as lysobisphosphatidic acid (LBPA) (Figure 1) [81,82]. Recently, TAT was shown to induce the leaky fusion of liposomes containing BMP [83]. TAT induces lipid mixing and membrane leakage in a BMP concentration-dependent manner and these activities were also greater at pH 5.5 than at physiological pH. This is important because pH 5.5 is characteristic of the acidic pH found in the lumen of late endosomes. The involvement of BMP in CPP-mediated delivery has not been demonstrated yet. However, cellular assays in which progression through the endocytic pathway is blocked with dominant negative Rab proteins have also suggested that TAT and polyarginine peptides only escape from the endocytic pathway upon reaching late endosomes [56]. Together, these results strongly suggest that BMP is a target for CPPs and that fusion between the limiting membrane of late endosomes and that of intraluminal vesicles is involved in the escape of CPP-cargo to the cytosolic space of cells.

## 3. Strategies to Improve Endosomal Release of CPP-Cargos

### 3.1. Multivalent CPPs

#### 3.1.1. Multivalency: Concept and Rationale for the Increase of CPPs Activity

**Figure 2:**
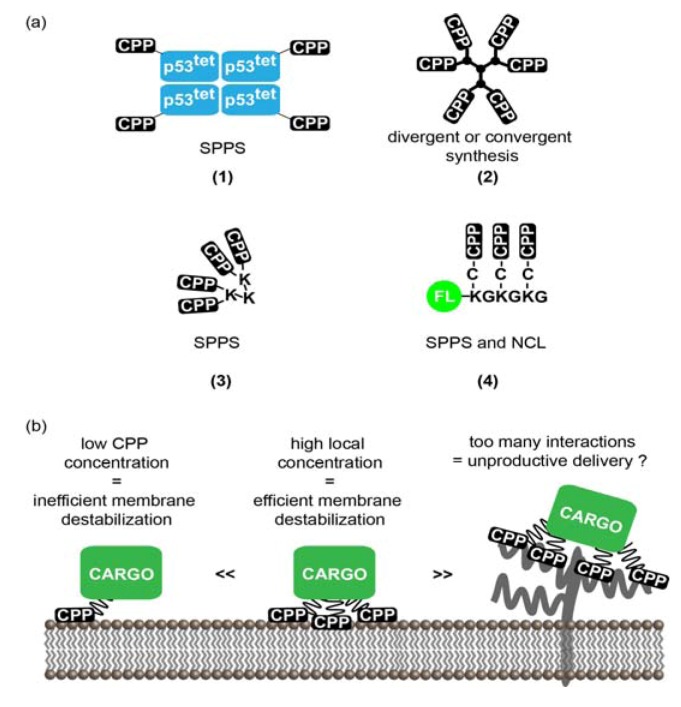
MCPPs systems and their interactions with membranes. (a) Strategies used to generate MCPPs. From left to right: (1) The p53^tet^-CPP system involves connecting the tetramerization domain from the human tumor repressor protein p53 to a CPP. The peptide sequence is generated using SPPS. After purification of the peptide sequence, p53^tet^ self-assembles into a tetramer producing a tetravalent CPP. (2) Loligomers are a “squid-like” MCPP system. The CPP is attached on the surface of a polylysine branch scaffold. The scaffold and the peptide are generated using SPPS. The number of branches of this MCPP system will depend on the number of Lys coupling steps. (3) The branched CPP system involves the generation of a peptide scaffold of Lys(ε-NH-Cys)Gly repeats to which a CPP-thioester is added using native chemical ligation (NCL). The peptides were generated using SPPS although production of recombinant peptide or protein thioesters is possible using intein fusions [100,101]. A fluorophore was added on the *N*-terminal end of the scaffold peptide to serve as both an imaging agent and a small model cargo. (4) Dendrimers are MCPPs having a “tree-like” shape and are usually generated using divergent or convergent methods. CPPs have been attached to the surface of polyamidoamine (PAMAM), polypropylenimine (PPI) or polyethylenimine (PEI) dendrimers. The number of CPPs on dendrimers is among the highest observed for MCPPs. (b) Possible model of mode of action of CPPs versus that of MCPPs. In this example, a CPP-cargo is present at the membrane of endosomes at a low local concentration, leading to poor endosomolytic activity. In contrast, a MCPP-cargo displays multiple copies of the CPP. This leads to efficient membrane interactions and a possible enhancement in membrane disruption. In some cases however, too many CPP copies might cause unproductive membrane interactions such as tight binding to HSPGs. This in turn might lead to poor cellular penetration.

One way to increase the endosomolytic activity of CPPs is to create multivalent CPPs (MCPPs). This approach consists of presenting multiple copies of a CPP on a delivery vector so as to increase the local concentration of the CPP where the peptide interacts with cellular components (Figure 2b). Several multivalent CPPs have been reported. Here, we survey the protocols used to generate MCPPs and discuss the evidence suggesting that multivalent CPPs escape from endosomes more efficiently than their monomeric counterparts. 

#### 3.1.2. Strategies to Generate MCPPs

Multiple synthetic protocols have been used to create MCPPs (Figure 2a). One strategy involves attaching a protein oligomerization domain to CPPs. For instance, the tetramerization domain of the human tumor suppressor p53 (p53^tet^), corresponding to amino acids 325–355, has been attached to the CPPs decaarginine and decalysine by solid phase peptide synthesis (SPPS) [84,85]. The p53^tet^ domain self-assembles in solution and tetrameric CPP constructs were therefore obtained (Figure 2a (1)). Notably, these compounds delivered DNA into cells more efficiently than decaarginine and decalysine alone. Multivalent CPPs have also been generated by attachment of the peptides to dendrimers [51,86,87,88,89]. Dendrimers are highly branched molecules that display a tree-like shape (Figure 2a (2)) [90]. The branches of dendrimers can be functionalized and, therefore, dendrimers can serve as a scaffold onto which CPPs can be chemically conjugated. Related approaches consist of attaching CPPs to peptide scaffolds. Branched “squid-like” peptide constructs known as loligomers have, for instance, been reported (Figure 2a (3)) [91,92,93]. Loligomers containing eight CPP copies have been assembled by SPPS on branched polylysine scaffolds [94]. TAT has also been assembled on a peptide scaffold consisting of Lys(ε-NH-Cys)Gly repeats (Figure 2a (4)). In this example, TAT was functionalized with a C-terminal thioester and conjugated to the cysteine residues of the scaffold by native chemical ligation [95,96]. The MCPPs generated displayed 2, 3, and 4 TAT copies. Finally, CPPs containing cysteine residues have been dimerized by the straightforward formation of disulfide bridges [97,98,99].

#### 3.1.3. MCPPs Mediated Delivery

How the extracellular and intracellular behavior of MCPPs compares to that of their monomeric counterparts is not clear. There is certainly growing evidence that MCPPs transduce cargos into cells more efficiently than CPPs. The MCPPs described in the previous paragraph have, for instance, been reported to deliver molecules such as DNA, proteins, peptides, polysaccharides or small organic fluorophores to a higher degree than CPPs [95,98,99,102,103,104]. However, in the case of DNA transfection experiments, improvements in cytosolic delivery can be difficult to interpret. Positively charged MCPPs form complexes with DNA that might be significantly different than those formed with CPPs. As a matter of fact, it has been proposed that branched CPPs might be more capable of “multitasking”, with a few branches of MCPPs being in complex with its DNA cargo while other branches might interact with cellular components [103]. In contrast, a positively charged and monomeric CPP bound to negatively charged DNA might not be able to interact with other molecules as readily.

Taking the example of DNA transfection again, MCPPs/DNA complexes are typically endocytosed at higher levels than CPPs/DNA particles [84,91,102]. Since more MCPPs and more DNA accumulate inside endosomes, more DNA is delivered into the cytosol and nucleus of cells. This would presumably happen even when MCPPs and CPPs might have similar intrinsic endosomal escape activity. It is therefore often difficult to determine whether increased delivery by MCPPs is due to enhanced endocytosis, enhanced endosomal release, or both. However, some experimental data support the notion that MCPPs might escape from endosomes more efficiently than monomeric CPPs. The fluorescently labeled trimeric peptide Fl-TAT_3_ has been shown to deliver molecules in cis (direct conjugation) or trans (simple co-incubation) to the cytosol of cells [95]. This activity was inhibited by amiloride, indicating that macropinocytosis is a required step in this process and that this MCPP releases molecules from the lumen of endocytic organelles. Notably, the monomer Fl-TAT and the dimer Fl-TAT_2_ failed to promote cytosolic delivery at concentrations five to ten times greater than that used for Fl-TAT_3._ At these higher concentrations, more Fl-TAT and Fl-TAT_2_ are endocytosed than Fl-TAT_3 _(unpublished data). Overall, the trimeric MCPP appears to escape from endosomes more efficiently than the monomeric or dimeric CPP. 

#### 3.1.4. Limitations and Future Challenges

MCPPs show great potential as endosomolytic compounds. However, several limitations of this class of compounds can be expected. First, synthesis protocols are often complex and MCPPs cannot be generated as easily as their monomeric counterparts. In addition, convenient protocols for the conjugation of these systems to various cargos remain to be established. The *in vivo* utility of MCPPs inside cells is also uncertain because of the potential immunogenicity of branched peptides in general. The possibility that CPPs might act as immunogens has already been discussed in the literature [13]. Notably, MCPPs share close structural resemblance with multiple antigenic peptides (MAPs) [105]. MAPs are known to induce strong immune responses in part because of enhanced molecular recognition by immune cells [106]. The latter is a result of the display of multiple short antigenic peptides in a polylysine dendrimer scaffold. The use of MCPPs could therefore produce a non-desired cellular immune response due to the display of multiple, possibly immunogenic CPPs. 

One of the challenges ahead consists in understanding how MCPPs mediate better delivery than monomeric peptides. To date, how the number of CPP copies impacts activity remains uncertain. As indicated in Figure 2b, few CPP branches might not induce enough activity while too many might cause unproductive behavior. The effect of cargo conjugation also remains to be explored. It is, for instance, possible that the number of CPP branches required for optimal delivery depends on the types of cargo used. Structure-activity relationship studies should also reveal how the architecture of MCPPs affects their interactions with lipid bilayers, their trafficking inside cells, and their endosomolytic activity. 

### 3.2. PMAPs (pH-Dependent Membrane Active Peptides)

The endosomal escape of CPP-cargo conjugates can be increased by the addition of peptides that disrupt membranes at acidic pH [35]. This approach is based on the fact that the pH in the lumen of endocytic organelles changes from neutral to acidic during endosomal maturation. Acidification is mediated by the vacuolar H^+^-ATPase proton pumps and pH gradually drops as maturation occurs from early endosomes (pH~6.5) to late endosomes (pH~5.5) and lysosomes (pH~4.5) (Figure 1) [107,108]. As endosomes acidify, endocytosed pH-sensitive peptides shift from an inactive state to an active, membrane-disruptive state. Therefore, in principle, these peptides are able to disrupt endosomal membranes without damaging the plasma membrane or membranes of other organelles. A class of such peptides is the pH-dependent membrane-active peptides (PMAPs). A prototypical example is the HA2 fusion peptide, a peptide that corresponds to the 23 N-terminal residues of the hemagglutinin HA2 subunit of the influenza virus X31 strain (GLFGAIAGFIENGWEGMIDGWYG) [109]. The influenza virus uses endosomal acidification as a triggering mechanism to deliver its genome into host cells [109,110]. The viral hemagglutinin undergoes a complex series of conformational changes to induce fusion of the viral and host membranes [111,112]. In the context of hemagglutinin, the HA2 peptide serves as an anchor that inserts into the endosomal membranes of host cells [113,114]. 

Interestingly, the HA2 peptide alone is able to fuse and lyse lipid bilayers in a pH-dependent manner [109,115]. The pH-dependent membrane-disruptive properties of PMAPs correlate with the protonation of glutamate or aspartate residues [109,115], and addition of glutamate residues has been shown to alter the pH at which HA2 analogues become active [116]. Protonation of glutamate and aspartate residues increases the net hydrophobicity of the peptide, and thus its propensity to bind or insert into lipid bilayers [117,118]. Upon insertion into membranes, PMAPs induce multiple events such as membrane fusion, leakage, and lysis [116,119]. The HA2 peptide and its analogues have been shown to be active on liposomal membranes [116,119,120] as well as on biological membranes. Several PMAPs have been developed, some of which are described herein (Table 1).

**Table 1:** Examples of PMAPs that increase endosomal escape of cargos.

Peptide	Sequence	Cargo	Ref.
HA2E5-TAT	GLFEAIAEFIENGWEGLIEGWYG	mCherry, Fluorescently labeled dextran	[121]
HA2-penetratin	GLFGAIAGFIENGWEGMIDGRQIKIWFQNRRMKWKK-amide	Penetratin:siRNA complex (50:1)	[122]
HA-K_4_	GLFGAIAGFIENGWEGMIDG-SSKKKK	Plasmid DNA, plasmid DNA+ lipofectamine™	[123]
GS-HA2: HA2-coated gelatin-silica nanoparticles (GSNP)	GDIMGEWGNEIFGAIAGFLGC (coating thru disulfide bond)	Plasmid DNA (pGL3)	[124]
	GS-TH: Tat and HA2-coated GSNP (coating thru disulfide bond)		
HA2E4	GLFEAIAGFIENGWEGMIDG GGYC	EGF-poly lysine and BODIPY-labeled antisense oligonucleotide (ONs) complex	[125]
Biotinylated TAT-HA2	(Biotin-CKYGRRRQRRKKRG-GDIMG EWGNE IFGAI AGFLG	Anti-biotin antibody coated gold nanoparticles	[126]
GALA	WEAALAEALAEALAEHLAEALAEALEALAA	siRNA, Nanoparticles	[127,128]
INF-7--(PEG)_6_-NH	GLFEAIEGFIENGWEGMIDG WYG-(PEG)_6_-NH_2_	Fluorescently labeled TAT-NeutrAvidin	[129]
GALA-INF3-(PEG)_6_-NH	GLFEAIEGFIENGWEGLAEALAEALEALAA-(PEG)_6_-NH_2_	Fluorescently labeled TAT-NeutrAvidin	[129]
GALA-INF3-(PEG)_6_-NH	GLFEAIEGFIENGWEGLAEALAEALEALAA-(PEG)_6_-NH_2_	Fluorescently labeled TAT-NeutrAvidin	[129]
INF-7	GLFEAIEGFIENGWEGMIDG WYG	Polyplex	[130,131,132]
diINF-7	GLFEAIEGFIENGWEGMIDG WYGC (dimerizing through Cys)	siRNA, DNA, immunoliposome encapsulated diphtheria toxin A chain (DTA)	[133,134,135]
INF7-SGSCG	GLFEAIEGFIENGWEGMIWDYG-SGSCG	Polyplex (pCMVLuc:K8)	[136]
INF7-K(GalNAc)_2_	GLFEAIEGFIENGWEGMIWDYG-SGSC-K(GalNAc)_2_	Polyplex (pCMVLuc:K8)	[136]

In their simplest form, PMAPs are composed of hydrophobic residues such as leucine or alanine as well as protonatable residues such as glutamate or aspartate. GALA (WEAALAEALAEALAEHLAEALAEALEALAA) is one such peptide. Upon acidification GALA takes on a helical conformation, with the hydrophobic residues on one face of the helix and the hydrophilic residues on the other. GALA has been shown to cause liposomal leakage in a pH-dependent manner [120]. It has been used successfully to deliver nanoparticles and siRNA to the cytosolic space of cells [127]. This delivery is also facilitated by conjugation of GALA to lipids [128].

Histidine residues can replace glutamates as endosomal escape facilitators, and have also been incorporated into PMAPs. Unlike glutamate, histidine becomes charged and therefore more hydrophilic upon protonation. Nevertheless, polyhistidine is known to disrupt membranes upon acidification and to enhance macromolecule release from endosomes [137,138,139], and this property has been applied to some PMAPs. The histidine-rich HA2 analogue H5WYG (GLFHAIAHFIHGGWH GLIHGWYG), for example, shows improved efficiency of gene transfer in cells [140,141]. Endo-Porter is a synthetic histidine-rich PMAP. Like GALA, this peptide preferentially forms a helical structure with a hydrophobic face consisting almost entirely of leucine, while the hydrophilic face consists of histidine residues. The lipophilic leucines help the peptide to bind membranes, which protonated histidines can then disrupt [142]. The exact sequence of Endo-Porter has not been reported. Endo-Porter is effective in delivering functional morpholino oligonucleotides to cultured cells and even whole kidneys [142,143,144].

Peptides of non-viral origin such as the antimicrobial peptide melittin have also been employed for endosomal disruption. Melittin (GIGAVLKVLTTGLPALISWIKRKRQQ) is a membrane-disruptive peptide which is derived from the venom of European honey bee *Apis* mellifera [145]. This peptide is active at neutral as well as acidic pH. It is therefore very toxic to cells on its own, as it can permeabilize the plasma membrane [146]. In order to confer a pH-dependent response to melittin, key lysine residues in the peptide have been masked with dimethylmaleic anhydride (DMMAn) [147,148,149,150]. DMMAn blocks the lytic activity of the peptide at the plasma membrane, but gets cleaved from the peptide at pH 5. The protected melittin is therefore unmasked and active in the acidified lumen of endosomes. DMMAn-melittin has been used to deliver siRNA and other nucleic acids to cells. A recent study shows that CM_18-_TAT_11_ (KWKLFKKIGAVLKVLTTG-YGRKKRRQRRR) can permeabilize endosomes and release a variety of co-incubated macromolecules into the cytosol [151]. In this approach, CM_18_, a hybrid peptide containing the first seven residues of cecropin-A and residues 2-12 of melittin, is not protected as reported with DMMAn-melittin. One might therefore expect that CM_18_-TAT_11_ might lyse various membranes and thereby cause damage to cells. However, the peptide is non-toxic at low micromolar concentrations. The authors have proposed that the lytic activity of CM_18_-TAT_11_ is expressed inside endosomes because the peptide reaches a critical membrane-disrupting concentration by accumulating in these organelles. In contrast, the peptide does not disrupt the plasma membrane of HeLa cells during incubation if its concentration is kept below this critical threshold (~8–16 μM). Notably, CM_18_-TAT_11_ also appears to remain mostly associated with lysed endosomes. While it is unclear why this is the case, it might prove advantageous as antimicrobial peptides that can enter cells are known to disrupt mitochondria and cause apoptosis [152,153]. Overall, this report therefore suggests that some antimicrobial peptides might be able to lyse endosomes without causing significant damage to other cellular membranes.

#### 3.2.1. PMAP-CPP Chimeras

PMAPs have been used in combination with CPPs to increase the endosomal escape of various cargos. This strategy is attractive because PMAP-CPP fusions can be genetically encoded or easily synthesized by SPPS. Chimeric hybrids of PMAPs and CPPs can therefore be readily obtained. HA2 derivatives are typically attached to the N-terminus of CPPs because HA2 requires a free N-terminal glycine for full membrane activity [154,155]. It has been shown that TAT-mediated delivery of cell-impermeable cargos into cells is enhanced by conjugation to PMAPs [156]. HA2-TAT and its derivatives have been attached to quantum dots, and such conjugates have been shown to stimulate macropinocytosis and endosomal escape in cultured cells [157]. Protein cargo delivery can also be enhanced by this method, and has potential for therapeutic applications. HA2 fused to the tumor suppressor protein p53 containing the poly-arginine CPP R11 (HA2-p53-R11) was, for instance, more efficient in abrogating cancer cell growth than p53-R11 alone [158]. While HA2 can be directly connected to the cargo, HA2-TAT has also been used in trans to increase the delivery of cargos co-incubated with cells. The principle is that HA2-TAT and a TAT-labeled cargo will accumulate together inside the endocytic pathway. HA2-TAT can then induce the endosomal escape of a TAT-cargo that would be present in the lumen of the same organelles. For example, a retro-inverso HA2-TAT, which is less susceptible to degradation within the cell, has been used to increase the delivery of TAT-Cre recombinase [156]. 

Interestingly, HA2-TAT derivatives can also increase the delivery efficiency of proteins that are not labeled by TAT or other CPPs. For instance, the derivative HA2E5-TAT has been used to deliver fluorescent proteins not labeled with a CPP to the cytosol of cells by simple co-incubation [159]. The apoptosis-inducing peptide PAD was also delivered to cells efficiently when co-incubated with cells, and successful cytosolic delivery was assessed by induction of cellular apoptosis. Amiloride inhibited PAD-mediated cell death in the presence of HA2E5-TAT, suggesting that HA2E5-TAT induces uptake of soluble cargo by macropinocytosis [159]. The strategy is therefore to take advantage of the fact that TAT can induce pinocytic uptake of cargo molecules that do not necessarily interact with cells but are simply present in the extracellular milieu. These cargo molecules, despite not containing TAT, also accumulate in the lumen of endocytic organelles that contain HA2-TAT.

#### 3.2.2. Mechanisms of PMAP-CPP Mediated Endosomal Escape

The fusion and lytic properties of HA2 and HA2 analogues have been extensively investigated *in vitro* because of their relevance to viral infection. The behavior of these compounds when attached to CPPs is, on the other hand, less well understood. Furthermore, the complex nature of the endosomal environment makes *in cellulo* mechanistic studies difficult, and much remains unknown about the pathway that PMAP-CPPs might follow and how endosomal escape is mediated on a molecular level. Nevertheless, inferences from liposome experiments, hemolysis data, and microscopy experiments in cultured cells can be made. 

Liposomes have been used to study the properties of INF7 (Table 1) [116]. Artificial lipid bilayers are a controllable model system of reduced complexity. Using such a system, INF7 was shown to induce both fusion and leakage in liposomes that was more active at reduced pH. Based on these results, one can extrapolate that HA2 derivatives might cause endosomal escape by inducing leaky fusion. Whether this phenomenon is actually dependent on pH remains to be established [116]. 

Red blood cells (RBCs) provide a model system that is more complex and physiologically relevant than liposomes, but significantly easier to work with than other cells. The release of hemoglobin from RBCs can be easily monitored, and RBCs typically do not fuse with one another during hemolysis assays [109]. The lytic activity of PMAP-CPPs can therefore be examined without interference from the fusion activity. Hemolysis by HA2E5-TAT has been examined and, as expected, HA2E5-TAT lyses RBCs more efficiently at low pH than at neutral pH. In particular, HA2E5-TAT, despite the presence of multiple glutamate residues, follows a single-protonation model. That is, the peptide appears to equilibrate between an active protonated form and an inactive deprotonated form. The protonation event has an apparent pKa of 6.7. HA2E5 alone follows a similar behavior. However, the apparent pKa of this peptide is 5.5 [71]. This therefore indicates that TAT can affect, and in this case increase, the pH-dependence and lytic activity of the HA2 moiety. 

**Figure 3:**
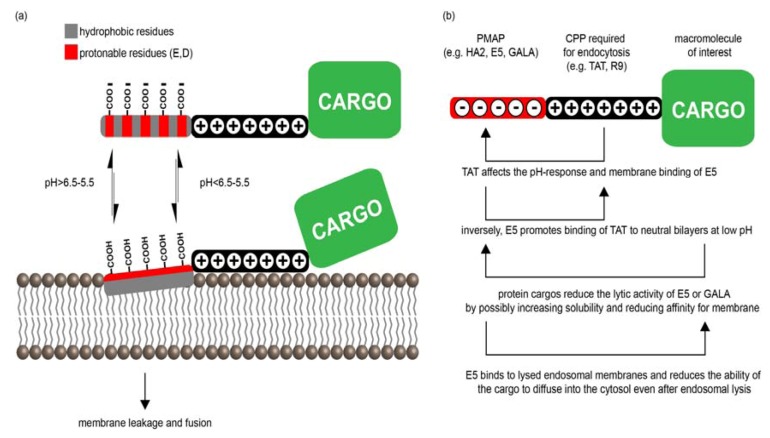
(a) PMAPs undergo conformational change upon acidification. This typically involves a shift from a random coil to an ordered conformation such as a helix, in which hydrophobic moieties are on one face while the ionizable moieties are on the other. This ordered conformation leads to membrane binding, and brings conjugated CPPs and cargos into close proximity to the membrane. At sufficient concentrations, this binding eventually leads to lysis or membrane fusion resulting in cargo release. (b) PMAPs, can interact with CPPs and cargo proteins. Most PMAPs are negatively charged in their deprotonated state and might interact electrostatically with positively charged CPPs. Soluble cargo proteins seem to reduce PMAP lytic activity, possibly by increasing the net hydrophilicity of the construct. PMAPs might also prevent release of cargos into the cytosol even after endosomolysis, possibly by tethering cargos to the ruptured membrane of endosomes.

The mechanism of HA2E5-TAT hemolysis was examined using a fluorescent analogue of the peptide [71]. HA2E5-TAT in its deprotonated form is soluble and interacts minimally with the membranes of RBCs. As pH is lowered and as the peptide becomes protonated, binding to membranes increases and this leads to more lysis (Figure 3a). This model is different than what has been proposed for HA2. Structural studies have suggested that HA2 is bound to the lipid bilayer at both pH 5 and pH 7 but that the peptide inserts into the lipid bilayer more deeply under acidic conditions [5]. This deep insertion has in turn been proposed to participate in the membrane disruptive activity of the peptide. 

Here again, it is possible that addition of the highly hydrophilic TAT could reduce the affinity of the peptide for the lipophilic environment of a membrane and alter its mode of action. Kinetic studies performed on single RBCs revealed that HA2E5-TAT-mediated hemolysis was a relatively slow event. Indeed, cells exposed to 5 μM peptide lysed over a period of approximately 90 sec [71]. These results indicate that rather than causing significant or widespread membrane damage, these peptides form only few pores in the membrane large enough for the 68 kDa hemoglobin to escape [160]. Overall, these studies suggest that in addition to inducing fusion, HA2 derivatives could potentially form pores in the membrane of endocytic organelles upon acidification of the endocytic pathway. 

#### 3.2.3. PMAP Interactions with CPPs and Cargos

Conceptually, PMAP-CPP chimeras are designed to contain two independent functionalities: the CPP is used to induce efficient endocytosis of a cargo while the PMAP is used to increase endosomal escape. However, CPPs, cargos, and PMAPs can interact with each other as well as with membranes, and several studies have highlighted how these interactions might affect intracellular delivery (Figure 3b). As previously stated, TAT affects the apparent pKa of HA2E5 [71]. TAT also binds negatively charged proteins such as bovine serum albumin (BSA) while HA2E5-TAT does not [159]. Overall, these results indicate that HA2E5 and TAT might interact with one another in a way that alters their properties. Given that PMAPs such as HA2E5 are rich in negatively charged glutamates and that TAT is rich in positively charged arginines and lysines, electrostatic interactions between the two moieties are likely. Because TAT binds to negatively charged phospholipids, it can help bring the PMAP moiety in proximity with appropriate membranes in a pH-independent manner [161]. Thus, the membrane lipid composition of endocytic organelles might influence the membrane-disruptive activity of the PMAP-TAT complex by affecting the ability of TAT to interact with the membrane. 

The hydrophobicity of PMAPs makes them poorly soluble and prone to aggregation, particularly as their concentration increases [162]. Addition of CPPs and other hydrophilic moieties to PMAPs helps to solubilize them and prevents aggregation [162,163]. For example, addition of oligolysine at the C-terminus of HA2 has been shown to increase HA2 solubility [163]. However, while decreased aggregation can yield a higher concentration of active peptide, increased solubility can also decrease the affinity of the constructs for membranes. This in turn might lead to a reduction in membrane permeabilization. For instance, conjugation of the soluble protein mCherry to HA2E5-TAT significantly reduced the hemolytic and endosomolytic activity of the PMAP-CPP [121]. Similarly, a GALA-antibody conjugate did not cause the release of calcein from liposomes as efficiently as GALA alone. The lytic activity of the conjugate was not restored by introducing a linker between the antibody and GALA, suggesting that proximity to the antibody was not what inhibited GALA activity [164]. In both cases, it appears that the addition of a cargo significantly decreases the activity of the PMAP-CPP tag. Whether this can be attributed solely to reduced membrane binding or whether the cargo also directly interferes with lysis remains, however, to be determined. 

While cargo can affect the activity of a PMAP tag, it is also clear that the PMAP affects the trafficking properties of a CPP-cargo conjugate. This was recently illustrated by an HA2-TAT-mCherry construct [121]. Fluorescence microscopy assays on live cells have shown that HA2E5-TAT-mCherry could induce endosomal release. Indeed, a fluorescent dextran used as a marker of fluid-phase uptake could be released into the cytosol of cells in the presence of the protein. However, the protein itself remained in a punctate distribution even after endosomal lysis. In contrast, mCherry was released into the cytosol when it was conjugated to HA2E5-TAT through a disulfide bridge that can be cleaved upon cytosolic access. TAT-mCherry could also escape endosomes when co-incubated with HA2E5-TAT. Together, these results suggest that HA2E5 is the major contributor to endosomal retention and presumably acts by binding the membrane of the lysed endosome, thereby tethering any attached cargo to the lysed endocytic vesicles. Finally, microinjected HA2E5-TAT-mCherry did not access the nucleus of cells while TAT-mCherry and mCherry did. Fluorescence imaging also suggested that the HA2E5-TAT-mCherry might interact with intracellular membranes. Overall, these results illustrate a possible inherent problem for these constructs. On one hand, lipophilicity of the PMAP is required for disruption of endosomal membranes. On the other hand, the same lipophilicity might alter trafficking and function of attached cargos. 

#### 3.2.4. Toxicity

Ideally, PMAPs should only become active at acidic pH so as to prevent disruption of the plasma membrane or membranes of intracellular organelles once cytosolic delivery has been achieved. However, PMAPs exist in equilibrium between protonated and deprotonated forms at any pH [118]. This can be illustrated by a modified version of the Henderson Hasselbach equation:

pH = pKa + log (inactive peptide/active peptide)

where inactive and active peptide is protonated and deprotonated, respectively. At any given pH a defined fraction of the peptides will be active. Thus, even at neutral pH, a sufficiently high concentration of total peptide can lyse RBCs [159]. Concentrations above ~5 μM HA2E5-TAT has been shown to be toxic to HeLa cells [121]. CPPs might, however, contribute to modulating the toxicity associated with PMAPs. As previously mentioned, CPPs can interact with HSPGs [165,166]. Heparan, an HS analogue that binds to CPPs such as TAT (Figure 1), has been shown to inhibit the binding of HA2E5-TAT to RBCs and concomitantly inhibit hemolysis. The effect of heparan on HA2E5-mediated hemolysis is, on the other hand, negligible [159]. It is therefore interesting to speculate that interactions between HA2E5-TAT and HSPGs could reduce access of HA2E5–TAT to the plasma membrane lipid bilayer. These interactions could therefore protect cells from the lytic activity of the PMAP-CPP conjugate. On the other hand, interactions between HA2E5-TAT and HS within the lumen of endosomes could inhibit endosomolysis.

#### 3.2.5. Future Challenges

PMAPs can be readily conjugated to CPPs as well as cargo proteins. In principle, they interact with membranes in a pH-dependent manner, allowing them to disrupt endosomal membranes only at appropriate concentrations. While they can enhance CPP-mediated delivery, PMAP-CPP conjugates present several challenges. How to incorporate PMAP-CPPs to achieve optimal delivery and minimal toxicity remains to be further explored. As illustrated before, how cargo conjugation affects PMAP-CPP activity remains in particular unclear. Endosomal retention of cargo fused to the PMAP-CPP construct, even after endosomal lysis, is a concern. This phenomenon seems to indicate that preferable designs should include cleavable linkers between PMAP-CPPs and their cargos. Such designs would, in principle, increase endosomal escape and cytosolic delivery.

### 3.3. Photochemical Internalization Using CPPs

Photosensitizers that accumulate in the endocytic pathway have been used to achieve endosomal release. Photosensitizers are compounds that produce reactive oxygen species (ROS) upon excitation with light. ROS can in turn damage membranes by reacting with membrane components and cause membrane permeabilization. Light can therefore be used as a trigger to induce the lysis of endosomes when these organelles are loaded with photosensitizers. This photo-induced delivery strategy was termed photochemical internalization (PCI) by Berg and co-workers, who introduced this concept in 1999 [167]. PCI has been successfully applied to the cytosolic delivery of immunotoxins, oligonucleotides and other therapeutic agents such as bleomycin and doxorubicin inside cells using PCI both *in vitro* and *in vivo* [168,169]. The photosensitizers (PS) that are commonly used are sulfonated tetraphenylporphyrins (TPPS2a or TPPS4a), sulfonated aluminum phthalocynanines (AlPcS2a) and chlorins (TPCS2a) [167,170,171,172,173].

#### 3.3.1. CPP-Mediated Photochemical Internalization

In 2004, two groups made the observation that fluorescently labeled CPPs could also escape from endosomes upon irradiation with visible light. Arginine-rich CPPs labeled with fluorescein were released from endocytic compartments upon irradiation with laser light at 488 nm. Similar endosomal release was observed when the peptide was labeled with Alexa-Fluor 633, incubated with cells and irradiated with 633 nm laser light [174]. This phenomenon has also been demonstrated when the CPPs are conjugated to proteins. For instance, the protein p53 labeled with the CPP R11 and with the fluorophore FITC was observed to accumulate in endosomes upon incubation with cells. Repeated fluorescence imaging at 480 nm by confocal laser scanning microscopy, however, led to a redistribution of the FITC conjugate into the cytosolic and nuclear compartments of cells [175]. The use of fluorescently labeled CPPs for PCI has been extended to the cytosolic delivery of RNA molecules. Ohtsuki and co-workers have fused an RNA binding protein U1A to the TAT peptide. This TatU1A construct was labeled with Alexa Fluor 546 or cyanine fluorophores at its C-terminus. The fluorescently labeled TatU1A construct could bind small hairpin RNA (shRNA) containing a U1A binding sequence and carry its cargo into the endocytic pathway of cells. Irradiation with light corresponding to the excitation wavelength of the fluorophores led to release of shRNA from endosomes into cytosol as measured by the cytosolic redistribution of fluorescence as well as by gene silencing mediated by the delivered shRNA. It was therefore shown that gene expression could be spatially and temporally controlled using light irradiation [176]. 

In addition to the fluorophores cited previously (i.e. xanthenes, Alexa fluor dyes, cyanines), CPP-photosensitizer conjugates have also been tested. For instance, aluminum phthalocyanine (AlPcS) has been attached to TAT to photo-induce endosomal release and cytosolic delivery [177]. Better uptake of the photosensitizer 5-[4-carboxyphenyl]-10,15,20-triphenyl-2,3-dihydroxychlorin (TPC) was also seen when conjugated to oligoarginine R7 [178]. The irradiation wavelength required to induce endosomal escape has to match the excitation spectra of the fluorophore and so far, light in the range of 480 to 670 nm has been successfully used [167,168,169,170,171,172,173,174]. When irradiation is performed on a confocal laser scanning microscope, the fluorophores have to be subjected to multiple light exposures to achieve significant endosomal release. Consequently, cytosolic redistribution typically happens on a time scale of minutes. In contrast, irradiation on an epifluorescence instrument can lead to cytosolic redistribution within milliseconds [179]. This has, for instance, been observed with TAT labeled with the fluorophore tetramethylrhodamine irradiated at 560 nm [179]. Presumably, these differences are due to the fact that while the whole cell is exposed to light when using epifluorescence, only small sub-cellular areas are exposed to light at once when a scanning laser is used. Together, these results nonetheless suggest that light can be dosed and that, unlike with other methods, endosomal release can in principle be precisely controlled. In addition, it appears that most if not all endocytic organelles containing Fl-CPP conjugates lyse if sufficient light exposure is applied. The overall yield of cytosolic delivery using this methodology can therefore be very high. 

#### 3.3.2. Mechanisms of CPP-Mediated PCI

The molecular mechanisms of CPP-mediated PCI are not completely understood. While it is clear that PS-CPP conjugates might induce endosomal lysis by generation of ROS [180], the fact that Fl-CPPs might be photolytic can seem surprising. Indeed, the fluorophores used to synthesize Fl-CPP conjugates are widely used for various cell biology applications and they typically do not damage cellular membranes [23,181]. However, fluorophores can generate singlet oxygen upon light excitation. This process involves energy exchange between fluorophores in their triplet excited state and dissolved molecular oxygen. The generated singlet oxygen,^ 1^O_2_, is very reactive and is well known to damage biomolecules. Unsaturated lipids or membrane proteins can, in particular, be oxidized by singlet oxygen and membranes can subsequently be destabilized [182]. The amount of singlet oxygen produced by fluorophores is typically low. Consequently, fluorophores alone are typically not photolytic. Conjugation to CPPs therefore appears to enhance this activity. 

**Figure 4:**
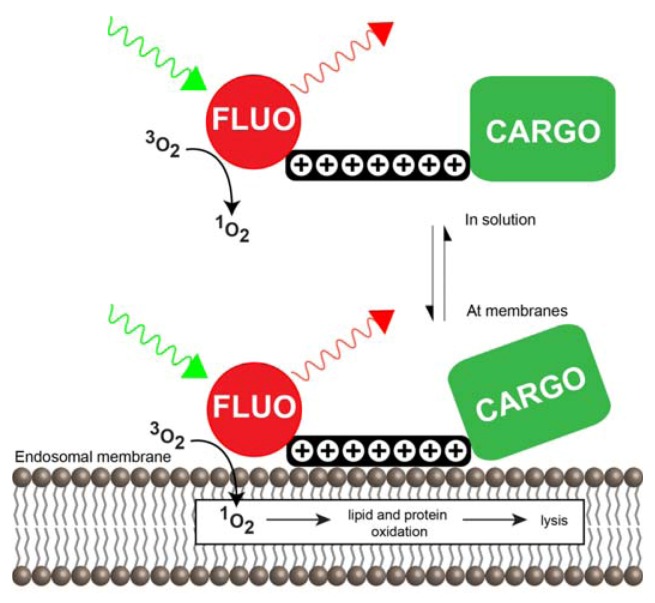
A model for fluorophore-CPP mediated PCI. The Fl-CPP conjugates exist in equilibrium in a membrane bound and unbound state due to the membrane-targeting role of the peptide moiety. Upon light irradiation, the fluorophore moiety of the conjugate produces singlet oxygen by transfer of energy from its triplet state to molecular oxygen. When present in solution, the Fl-CPP conjugate is innocuous. But, in its membrane bound state, the singlet oxygen produced by the fluorophore reacts with membrane biomolecules and disrupts the membrane, causing release of endosomal contents.

Recent evidence suggests that the photolytic activity of the Fl-CPP TMR-TAT requires the production of singlet oxygen. The singlet oxygen inhibitor crocetin has, for instance, been shown to reduce the light-induced endosomal escape of TMR-TAT inside cells [179]. However, singlet oxygen quenchers such as α-tocopherol inhibit photolysis of RBCs [179]. Together, these results support the notion that Fl-CPPs produce singlet oxygen inside endosomes upon light irradiation. While this has not been demonstrated yet, subsequent photo-oxidation of membrane components is likely involved in the endosomal escape of Fl-CPPs. A model for how Fl-CPP mediated photochemical internalization might work is depicted (Figure 4).

While required for lysis, production of singlet oxygen cannot solely explain the photo-endosomolytic activity of Fl-CPPs. For instance, a nona-lysine peptide labeled with TMR, TMR-K9, does not lyse endosomes upon light irradiation while TMR-TAT and TMR-R9 do [179]. This is true when, based on fluorescence intensity, TMR-K9 is present inside endosomes at higher levels than TMR-TAT. This is also true when cells containing TMR-K9 are exposed to more light than cells containing TMR-TAT. These results therefore indicate that the photo-endosomolytic activity of Fl-CPPs is not simply caused by the accumulation of singlet oxygen-generating fluorophores inside endosomes. *In vitro* photo-hemolysis assays have also confirmed that while TMR-TAT lyses RBCs readily, TMR-K9 does not [179].

It therefore appears that the CPP plays a direct role in transforming fluorophores into effective photolytic agents. What this role is remains unclear. However, singlet oxygen, as an excited state of oxygen, is known to have a short lifetime (< 4 µs) and a small diffusion distance (220 nm) [182]. This suggests that, in order for ^1^O_2_ to damage a membrane, its production should be in the vicinity of the target membrane. CPPs could therefore play the role of bringing the singlet oxygen-generating fluorophore in close proximity to endosomal membranes. Future studies should reveal whether this is the case or not. 

#### 3.3.3. Cell Death

A potential limitation of PCI and CPP-mediated PCI in particular is the photo-toxicity associated with these methodologies. The photosensitizers used for PCI can cause cell death when exposed to sufficient light [183]. The light dose applied to achieve endosomal release must therefore carefully be controlled so as to avoid cell death during delivery [183]. Several reports have shown that CPP-mediated PCI can be accomplished without killing cells [174,184]. However, R7-TPC causes apoptosis and necrotic cell damage at low and high concentrations, respectively [178]. Recently, rapid cell death was also found to occur during PCI with TMR-TAT. In particular, plasma membrane permeabilization and blebbing were observed shortly after endosomal escape of TMR-TAT was initiated with light [185]. 

It is clear that delivering photolytic agents into the cytosol of cells can potentially have detrimental effects. One can imagine how a photolytic Fl-CPP could, upon endosomal escape, reach the membrane of other intracellular organelles, damage these membranes during irradiation, and cause cell death. However, TMR-TAT is not phototoxic when directly microinjected into the cell’s cytosol [185]. Instead, endosomal lysis itself has been implicated in the cell death that accompanies PCI with TMR-TAT. In particular, irradiation causes the endosomal release of not only TMR-TAT but also of other molecules present in the lumen of these organelles [185]. The release of toxic material such as lysosomal hydrolases [171] or iron [186] might in principle take place. Recently, the release of calcium from endocytic vesicles photolysed with TMR-TAT has been implicated in cell-death. Release of calcium into the cytosol was followed by accumulation of calcium in the mitochondria, activation of the mitochondrial permeability transition pore (MPTP), and subsequent cell death [185]. Consequently, in tissue culture assays, rapid cell death was abolished with ruthenium red or cyclosporine A, inhibitors of mitochondrial calcium transport or of the MPTP, respectively [185]. 

#### 3.3.4. Future Challenges

In principle, CPP-mediated PCI could be used *in vivo* to deliver drugs to diseased cells by shining light only on selected tissues. PCI is particularly attractive for such applications because of the temporal and spatial control that it provides [176]. The *in vivo* potential of PCI has been demonstrated with photosensitizers [187,188,189]. However, CPP-mediated PCI has never been tested *in vivo* to our knowledge. Yet, one can envision how CPP-based agents might be complementary to other PCI photosensitizers. While peptides are prone to rapid degradation *in vivo* and *in cellulo*, retro-inverso (ri) peptides can be used to extend the half-life of Fl-CPPs. It has been observed that endocytosed TMR-TAT loses its photo-endosomolytic activity after only a few hours of incubation, presumably because of proteolytic degradation of the peptide within endocytic organelles [136]. In contrast, TMR-riTAT remains active even after 8 h of incubation [179]. Another important issue for *in vivo* applications is related to the fact that visible light does not penetrate tissues deeply [190]. It is reported that red and near-infrared light penetrates tissues more deeply than light of shorter wavelengths [190]. Chromophores that absorb light above the 600 nm range should therefore be preferable for *in vivo* procedures. However, Fl-CPPs conjugates containing Cy5, Cy5.5, Alexa 660, and Alexa 680 were reported to be dramatically less efficient at inducing the endosomal release of shRNA than fluorophores with shorter excitation wavelength [176]. Whether Fl-CPP can be performed with red or near-infrared chromophores therefore remains to be established.

## 4. Conclusions

It has become increasingly clear that endosomal entrapment is a major bottleneck in many cellular delivery methodologies, including CPP-mediated delivery. As highlighted in this review, several approaches can be exploited to improve the endosomal release of molecular cargos. Each of these approaches has advantages. Multivalent CPPs are efficient and can act as multitasking molecules. PMAP-CPP-cargo can be genetically encoded, allowing for relatively easy recombinant production. This approach is also advantageous if the cargo of interest is a protein or a peptide. PCI provides control over when and where cargo is released from endosomes. This may aid in cell or tissue specificity in *in vivo* applications. It may also be useful in cellular studies that require a known “time zero” of endosomal release. Despite these various advantages, these methodologies also have limitations. For instance, the *in vivo* potential of these strategies remains uncertain. The efficiency with which endosomal release is achieved is also far from being optimal. 

Endocytic organelles constitute a complex and dynamic environment where the activity of CPPs and other endosomolytic moieties can be modulated by interactions with many different binding partners. A better understanding of what interactions promote or inhibit endosomal release should be useful for the design of future drug delivery tools. It is also currently unclear as to which endocytic organelles might be disrupted by endosomolytic agents. In principle, the answer to this question will aid in optimizing the cytosolic delivery of intact, functional cargo. Indeed, one of the challenges associated with utilizing the endocytic pathway as a cellular gateway is to avoid degradation of molecular cargos that are sensitive to either the acidic pH or hydrolytic enzymes present in endocytic organelles. TAT-protein conjugates have, for instance, been shown to escape from endosomes after partial degradation [28]. Ideal endosomolytic reagents should therefore promote escape before degradation of the cargo takes place. In this regard, one can expect that endosomal release from endocytic vesicles or early endosomes should be preferable to release from late endosomes or lysosomes as these organelles are both more acidic and greatly enriched in degradative enzymes. 

An improved understanding of which organelles are disrupted by endosomolytic agents may also help to understand how delivery affects cells. One can envision how endosomolytic compounds might do more than simply increase overall cytosolic delivery. The cell-death observed with TMR-TAT-mediated PCI, for instance, highlights how extensive endosomal lysis might be toxic to cells. These results then raise the question of how endosomal lysis might impact the overall physiology of cells. Even when cell death is not observed, important cellular processes may be altered during delivery. In drug delivery applications where the goal is not to kill cells, it would presumably be preferable to use endosomolytic agents that can escape from endosomes with their drug cargos with only minimal side effects. Thus, understanding how to make CPPs and endosomolytic agents more efficient might only be a first step in designing ideal CPP-based drug delivery tools.
